# Population-Specific gene expression profiles in prostate cancer: insights from Weighted Gene Co-expression Network Analysis (WGCNA)

**DOI:** 10.1186/s12957-024-03459-6

**Published:** 2024-07-05

**Authors:** Laleh Manouchehri, Zahra Zinati, Leyla Nazari

**Affiliations:** 1 School of Medicine, Alma Mater Studiorum, Università Di Bologna, Via Zamboni, 33, 40126 Bologna, Italy; 2 Department of Agroecology, College of Agriculture and Natural Resources of Darab, Shiraz University, Shiraz, Iran; 3 Crop and Horticultural Science Research Department, Fars Agricultural and Natural Resources Research and Education Center, Agricultural Research, Education and Extension Organization (AREEO), Shiraz, Iran

**Keywords:** LASSO regression, Correlation, Feature selection, Transcriptome

## Abstract

This study investigates the genetic factors contributing to the disparity in prostate cancer incidence and progression among African American men (AAM) compared to European American men (EAM). The research focuses on employing Weighted Gene Co-expression Network Analysis (WGCNA) on public microarray data obtained from prostate cancer patients. The study employed WGCNA to identify clusters of genes with correlated expression patterns, which were then analyzed for their connection to population backgrounds. Additionally, pathway enrichment analysis was conducted to understand the significance of the identified gene modules in prostate cancer pathways. The Least Absolute Shrinkage and Selection Operator (LASSO) and Correlation-based Feature Selection (CFS) methods were utilized for selection of biomarker genes. The results revealed 353 differentially expressed genes (DEGs) between AAM and EAM. Six significant gene expression modules were identified through WGCNA, showing varying degrees of correlation with prostate cancer. LASSO and CFS methods pinpointed critical genes, as well as six common genes between both approaches, which are indicative of their vital role in the disease. The XGBoost classifier validated these findings, achieving satisfactory prediction accuracy. Genes such as APRT, CCL2, BEX2, MGC26963, and PLAU were identified as key genes significantly associated with cancer progression. In conclusion, the research underlines the importance of incorporating AAM and EAM population diversity in genomic studies, particularly in cancer research. In addition, the study highlights the effectiveness of integrating machine learning techniques with gene expression analysis as a robust methodology for identifying critical genes in cancer research.

**Supplementary Information:**

The online version contains supplementary material available at 10.1186/s12957-024-03459-6.

## Introduction

Prostate cancer is a substantial public health concern within the field of oncology due to the notable disparities observed in its incidence and progression across various population groups. African American men (AAM), specifically, exhibit a higher incidence rate and a higher probability of receiving advanced-stage diagnoses compared to men of other groups, thereby demonstrating a disproportionate impact [1]. Prior studies have indicated that genetic factors may contribute to the susceptibility and severity of prostate cancer, with particular gene mutations or variants being more common in distinct ethnic groups [2–4]. An example of a genetic variation in the NEDD9 gene has been discovered, which is closely linked to a higher likelihood of developing prostate cancer in individuals of African population. This genetic variant causes the overexpression of the NEDD9 gene, facilitating the onset and advancement of prostate cancer [3]. In addition, a recent study has investigated the connections between genetic variations in 30 alternatively spliced genes with the risk, aggressiveness, and survival rates of prostate cancer in both white and African-American populations. The study revealed that variations in single-nucleotide polymorphisms of genes that are alternatively spliced and connected to population descriptors are linked to the risk, aggressiveness, and survival of prostate cancer [2]. Nevertheless, the mechanisms contributing to the elevated occurrence and severity of prostate cancer in African Americans have not been definitively determined. Possible factors include socioeconomic status, biological aggressiveness, family history, and variations in genetic susceptibility [5–8].

From a biological standpoint, evidence indicates inherent variations in tumor features among different population groups. These variances may manifest themselves as varying rates of tumor growth or aggressiveness, requiring a more thorough investigation of hereditary factors. Nevertheless, the historical under-representation of minority groups in clinical trials and research studies impedes the development of a comprehensive understanding of cancer, thereby restricting the ability to gain insights into the disease’s behavior among diverse populations. Powell and his colleagues [9] discovered clear differences in the patterns of gene activity in prostate cancer between African-American men (AAM) and European-American men (EAM). Their examination of 639 tumor samples unveiled noteworthy group-specific disparities: A total of 95 genes had increased expression levels in AAM samples, whereas 132 genes displayed elevated expression levels in EAM samples. These findings emphasize the significance of considering population diversity in the field of cancer genetics and the necessity for tailored treatment strategies [2]. Recent advancements in the field of machine learning, have demonstrated the effectiveness of machine learning models in identifying gene biomarkers associated with prostate cancer [10, 11]. These authors employed various machine learning algorithms, including hierarchical clustering and support vector machines, to accurately classify the different stages and locations of prostate cancer. Using these models, relevant biomarkers were identified. These biomarkers significantly contribute to the understanding the molecular mechanisms underlying prostate cancer and provide a basis for more personalized and effective treatments.

The objective of this research is to address these gaps in knowledge and investigate the genetic foundations of these discrepancies through the utilization of Weighted Gene Co-expression Network Analysis (WGCNA) on microarray data obtained from prostate cancer patients belonging to two separate population cohorts. The motivation for doing such an analysis is based on the complex nature of the inequality observed in prostate cancer. Through the analysis of microarray data, our goal is to discern gene expression patterns and possible biomarkers that exhibit notable differences among the population groups under investigation. This method not only enhances the overall comprehension of disparities in prostate cancer, but it also facilitates the development of more customized and efficient diagnostic and therapeutic strategies, with the ultimate goal of diminishing the impact of prostate cancer on all groups.

## Materials and methods

**Fig. 1:**
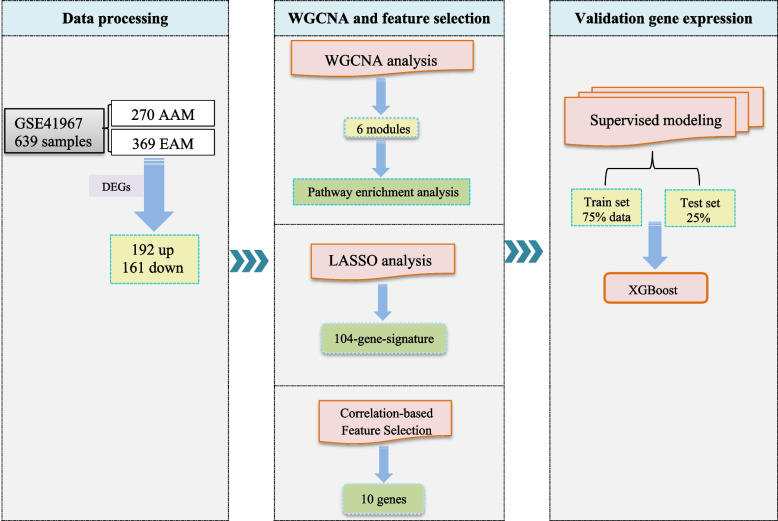
Flowchart of the study to find the key genes responsible for disparities in prostate cancer between African-American Men (AAM) and European-American Men (EAM)

Flowchart of the study to find the key genes involved in prostate cancer in European-American Men (EAM) and African-American Men (AAM) is presented in Fig. 1.

### Data acquisition and preprocessing

In this study, publicly available microarray gene expression data (GSE41967) was retrieved at Gene Expression Omnibus (GEO, http://www.ncbi.nlm.nih.gov/geo/). The study utilized primary tumor samples from the Gene Expression Omnibus (GEO) dataset (GSE41967) from prostate cancer patients [9]. The dataset included 270 African American men (AAM) and 369 European American men (EAM) collected from the Wayne State University (WSU) pathology core in Detroit, Michigan during 1991 to 1996. No samples were excluded, and all 639 samples were analyzed. Gleason's grade of the tumor was recorded with tumors stratified into aggressive (grades 7(4 + 3), 8, 9, and 10) and non-aggressive (grades ≤ 7(3 + 4)) categories. The dataset did not provide data on either metastatic disease or detailed geographical information about the individual patients and focused solely on primary tumors. The platform used for the gene microarray was GPL16230. The raw data were received and read into the R statistical environment (v. 4.1.2) using the GEOquery package (v. 2.62.2). The expression matrix was divided into AAM and EAM groups. A non-paired t-test provided by Limma [12] was used to find differentially expressed genes (DEGs). *P*-values < 0.01 were chosen as the threshold for the identification of DEGs.

### Weighted Gene Co-expression Network Analysis (WGCNA)

The fundamental basis of our scientific approach relied on WGCNA, a systems biology technique employed to identify clusters (modules) of genes that exhibit strong correlations. By employing this method, we were able to create a scale-free network that accurately depicts the complex patterns of gene expression connections found in the prostate cancer data. Using this network, we were able to detect groups of genes that have similar patterns of expression. These gene modules were then compared with the population backgrounds of the patient samples to find any correlations. The modules are deemed statistically significant based on their correlation coefficients and p-values. This phase was essential in identifying precise gene clusters that may have a significant impact on the differences reported in prostate cancer among different population groups.

The WGCNA R library (v. 1.71) [13] was conducted across the DEGs profile, including 353 genes. The gene dendrogram was employed for module detection by the dynamic tree cut method (minimum module size = 20, cutting height = 0.85, and deepSplit = 2). For network construction, the selected power (β) was set to 5. Module membership (MM) and gene significance (GS) were generated for selected modules. The hub-ness of a gene in each module was identified through the “chooseTopHubInEachModule” function.

### Pathway enrichment analysis

We conducted a pathway enrichment analysis using the DAVID database (https://david.ncifcrf.gov/) to obtain a better understanding of the significance of the selected modules in terms of pathways. The pathways were considered significant according to *p* < 0.01. The ggplot2 package (v. 3.3.6) was used to visualize the pathway enrichment analysis of the selected modules. This research facilitates comprehension of the various mechanisms by which the discovered genes may contribute to the observed differences in prostate cancer among different population groups.

### Least absolute shrinkage and selection operator

LASSO is a regularization technique developed by Tibshirani [14] to improve feature selection. A subset of informative features is selected by shrinking the regression coefficients to zero in the linear regression model. LASSO performs L1 regularization resulting in sparse models with few coefficients. The larger the penalties, the closer the coefficients are to zero, resulting in a simpler model. The purpose of the LASSO algorithm is to minimize the sum of squares of the error:

1 $$\sum_{i=1}^{n}{{(y}_{i}-\sum_{j}{x}_{ij}{\beta }_{j})}^{2}+ \lambda \sum_{j=1}^{p}\left|{\beta }_{j}\right|$$

In the equation, some coefficients of β are shrunk to zero; therefore, the output model is easier to interpret. The tuning parameter λ controls the L1 penalty strength. When λ equals zero, no feature is removed from the model. When λ increases, more coefficients are removed. As a rule, as λ increases, the degree of bias also increases. On the other hand, variance decreases with increasing λ.

To select cancer-responsive gene combinations reliably associated with prostate cancer, we used the R package *glmnet* (Version 4.1.4) [15] to fit a logistic LASSO regression model on the 353 DEGs, which were all included in the modules categorized by WGCNA. Here, we performed tenfold cross-validation using the ‘cv.glmnet’ function, and parameters were set as alpha = 1, family = "binomial".

### Correlation-based feature selection

Correlation based Feature Selection (CFS) is a filter method that measures the correlation between two nominal features. It is a fully automatic algorithm, without imposing any thresholds or limits on the number of selected features. Redundant features that might be highly correlated with other features are screened out. The acceptance of a feature will depend on its ability to predict classes in areas of the instance space not already predicted by other features. The CFS function is calculated as:

2 $${\text{Merit}}_{\text{s}}=\frac{k\overline{{r }_{cf}}}{\sqrt{k+k(k-1)\overline{{r }_{ff}}}}$$

In the above equation, Merit_s_ is the heuristic merit of a subset (s) of k features, r_cf_ stands for the average feature class correlation, and r_ff_ is the average inter-correlation of features. The Eq. 2, is Pearson’s correlation between standardized variables [16].

In this study, CFS was performed using Waikato Environment for Knowledge Analysis (WEKA) version 3.7.4 using the BestFirst search method. The space of attribute subsets was searched with default parameters (direction = Forward; LookUpCasheSize = 1; and SearchTermination = 1).

### Validation and reproducibility

In order to guarantee the strength and reliability of our results, we utilized stringent cross-validation methods and assessed the performance of XGBoost, a gradient boosting method incorporating the regression tree [17] to classify AAM and EAM samples based on the blue and yellow modules. XGBoost combines weak learners to create a single strong learner. Package ‘xgboost’ version 1.6.0.1 was used for the classification and Ckmeans.1d.dp version 4.3.4 was used for the importance ranking of the selected features. Cross validation XGBoost model was conducted, splitting the data into 75% training and 25% testing.

## Results

All samples were divided into AAM and EAM and submitted to the Limma package to find differential genes between the two groups. We found 353 DEGs, which 192 were up and 161 were down regulated in AAM in contrast to EAM. The expression matrix of these 353 DEGs was considered as input for WGCNA analysis.

### Weighted Gene Co-expression Network Analysis (WGCNA)

**Fig. 2:**
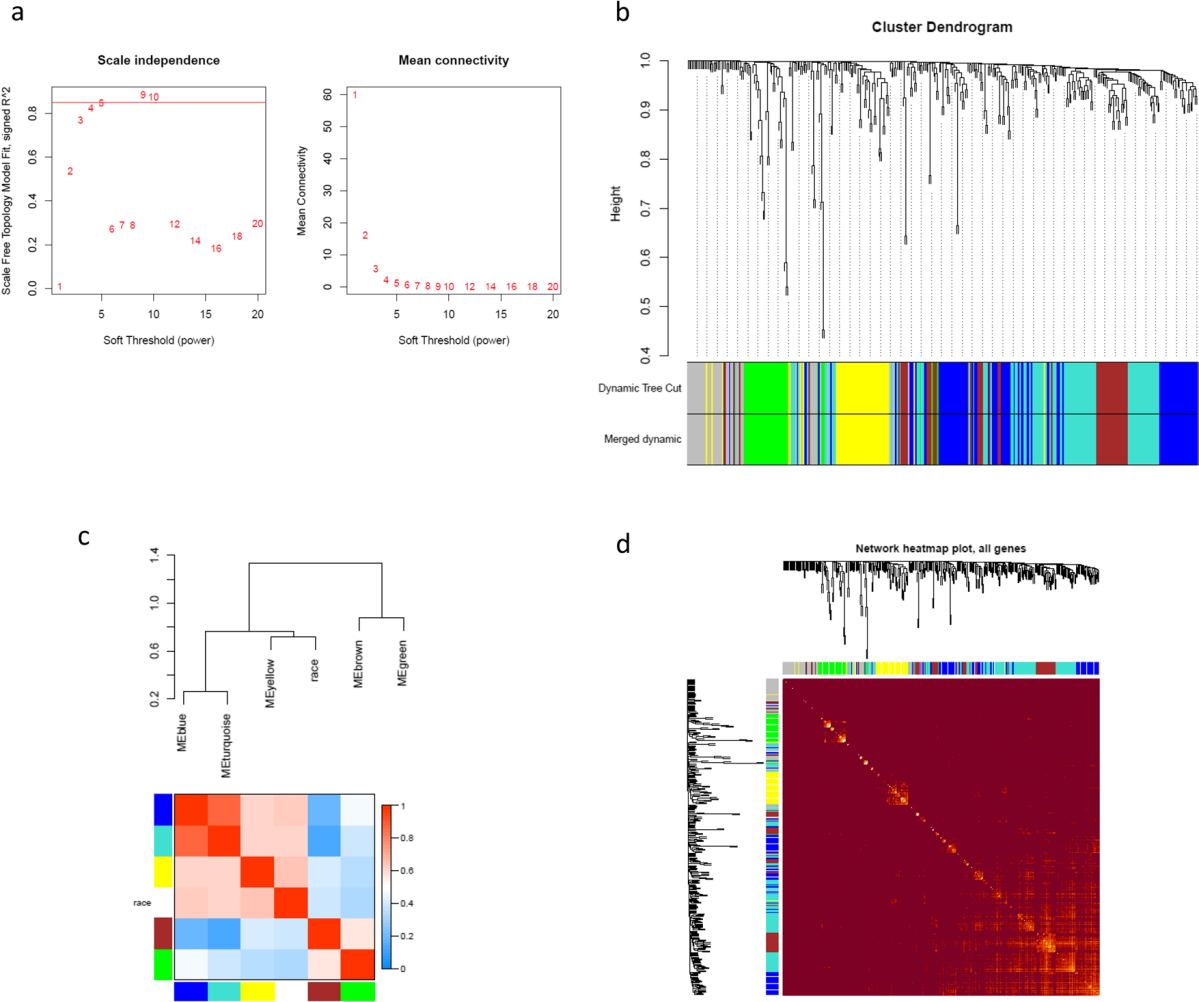
Analysis of network topology for various soft-thresholding powers (**a**). The left and right plots display the scale-free fit index and mean connectivity (y-axis), respectively, as a function of the thresholding power (x-axis). Gene dendrogram obtained by average linkage hierarchical clustering (**b**). The color row underneath the dendrogram shows the module assignment determined by the Dynamic Tree Cut and merged dynamic. Hierarchical clustering and heatmap plots of module eigengenes (**c**). Each row and column in the heatmap corresponds to one module eigengene (labeled by color) or weight. In the heatmap, blue color represents low adjacency (negative correlation), while red represents high adjacency (positive correlation). Network heatmap plot (**d**). Branches in the hierarchical clustering dendrograms correspond to modules displayed in the color bars below and to the right of the dendrograms. High co-expression blocks of interconnected genes are indicated by lighter colors. Genes with high intramodular connectivity are located at the tip of the module branches since they display the highest interconnectedness with the rest of the genes in the module. Dark color denotes low topological overlap, and progressively lighter red denotes higher topological overlap. Lighter squares along the diagonal correspond to modules

The connectivity graph, depicted in the right plot in Fig. 2a, demonstrates the correlation between the power of soft-thresholding and the scale independence of the network. The left plot in Fig. 2a displays the relationship between the scale-free topology model fit (R^2^) and several soft-thresholding powers. A greater R^2^ value signifies superior adherence to the scale-free topology, which is a characteristic feature of resilient biological networks. Our analysis identified an optimal soft-thresholding power (5) where the network achieves high-scale independence while maintaining a moderate level of mean connectivity. This threshold guarantees that the network achieves an optimal balance between sparsity and density, thereby enabling precise identification of gene modules through their co-expression patterns.

The cluster dendrogram (Fig. 2c) depicts the hierarchical clustering of genes. The vertical axis of the dendrogram reflects the dissimilarity metric, which indicates the degree of difference across gene modules. Every branch in the dendrogram corresponds to a specific gene, and the point at which branches combine indicates the level of similarity between the corresponding gene expression profiles. The dynamic tree cut exhibited in the dendrogram revealed the presence of multiple unique clusters. These clusters denote collections of genes with analogous expression patterns. These modules are particularly intriguing because they may contain genes that have essential functions in the distinct development or advancement of cancer in different population groups. A total of 353 DEGs out of the 639 applied to construct a dendrogram resulted in the identification of 6 modules based on average dynamic tree clipping and hierarchical clustering (Fig. 2b).

The network TOM plot has been visualized in Fig. 2d, where the *x*-axis and y-axis correspond to the logarithm of whole network connectivity and the corresponding frequency distribution, respectively. In this plot, modules are formed separately as 'fingers', and genes with high intramodular connectivity are located at the tips of the module branches.

### Module-Trait relationship analysis

The module-trait association is the most pivotal part of our findings, as it establishes a correlation between gene modules and distinct populatio groups.

**Fig. 3:**
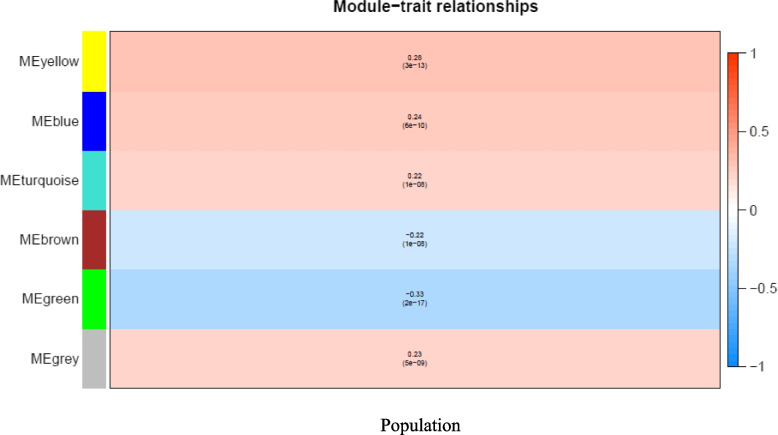
Consensus network modules correlated with population in European-American Men (EAM) and African-American Men (AAM). Correlation coefficients along with *p*-value in parenthesis underneath are presented. The legend at right is modules correlated to the population

The research revealed six modules that have a robust positive or negative connection with AAM and EAM. This suggests that these genes may have a substantial impact on the observed population differences in prostate cancer (supplementary Sheet 1 and 2). These modules were visualized in Fig. 3. The yellow (*r* = 0.28, *p* = 3 × 10^−13^) and green (*r* = -0.33, *p* = 2 × 10^−17^) modules were most positively and negatively correlated, respectively, with prostate cancer. Subsequently, six co-expression modules were clustered, with the blue module having the strongest similarity to the turquoise module (Fig. 3).

### Pathway enrichment analysis

**Fig. 4:**
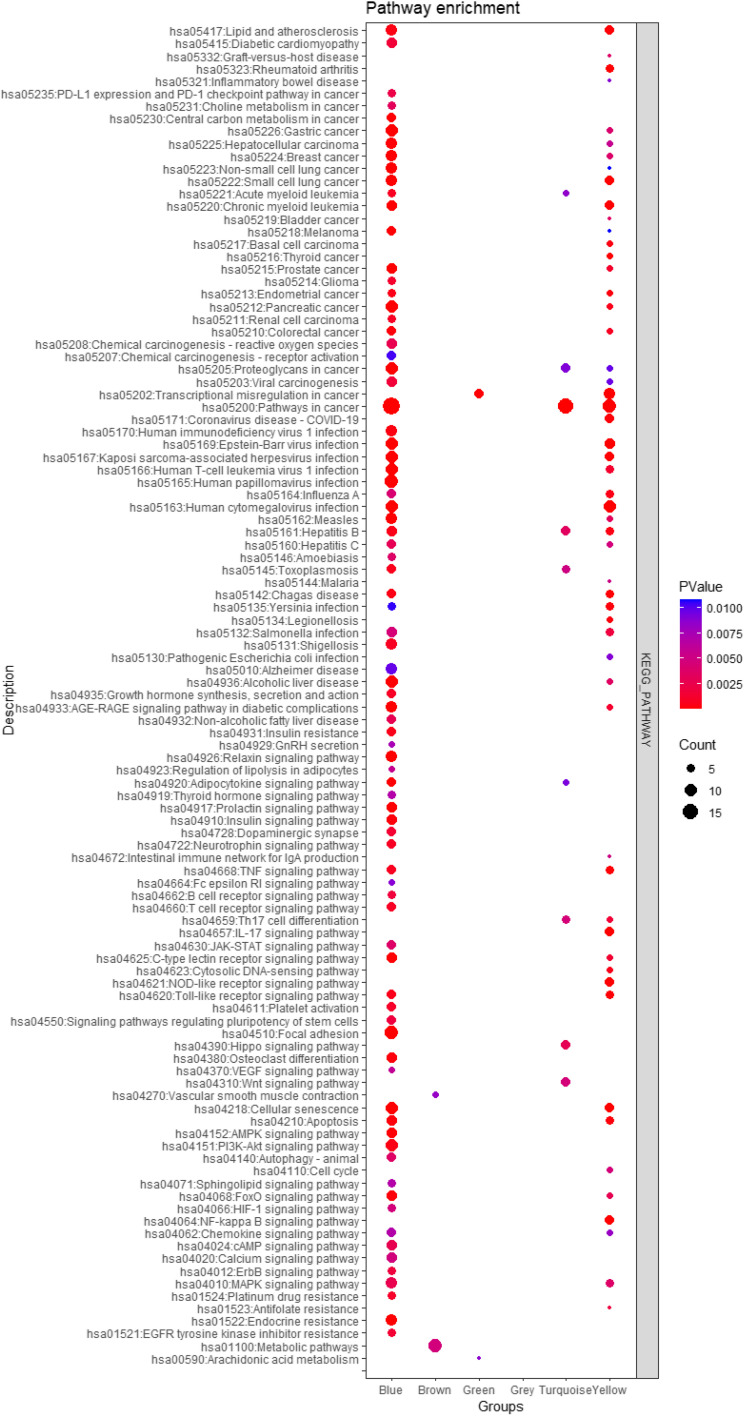
Pathway enrichment analysis of major modules obtained through weighted gene co-expression network analysis (WGCNA) in prostate cancer between European-American Men (EAM) and African-American Men (AAM)

Figure 4 presents the pathway enrichment analysis of major modules. The blue and yellow modules exhibit a strong correlation with the prostate cancer pathway.

### Least absolute shrinkage and selection operator

**Fig. 5:**
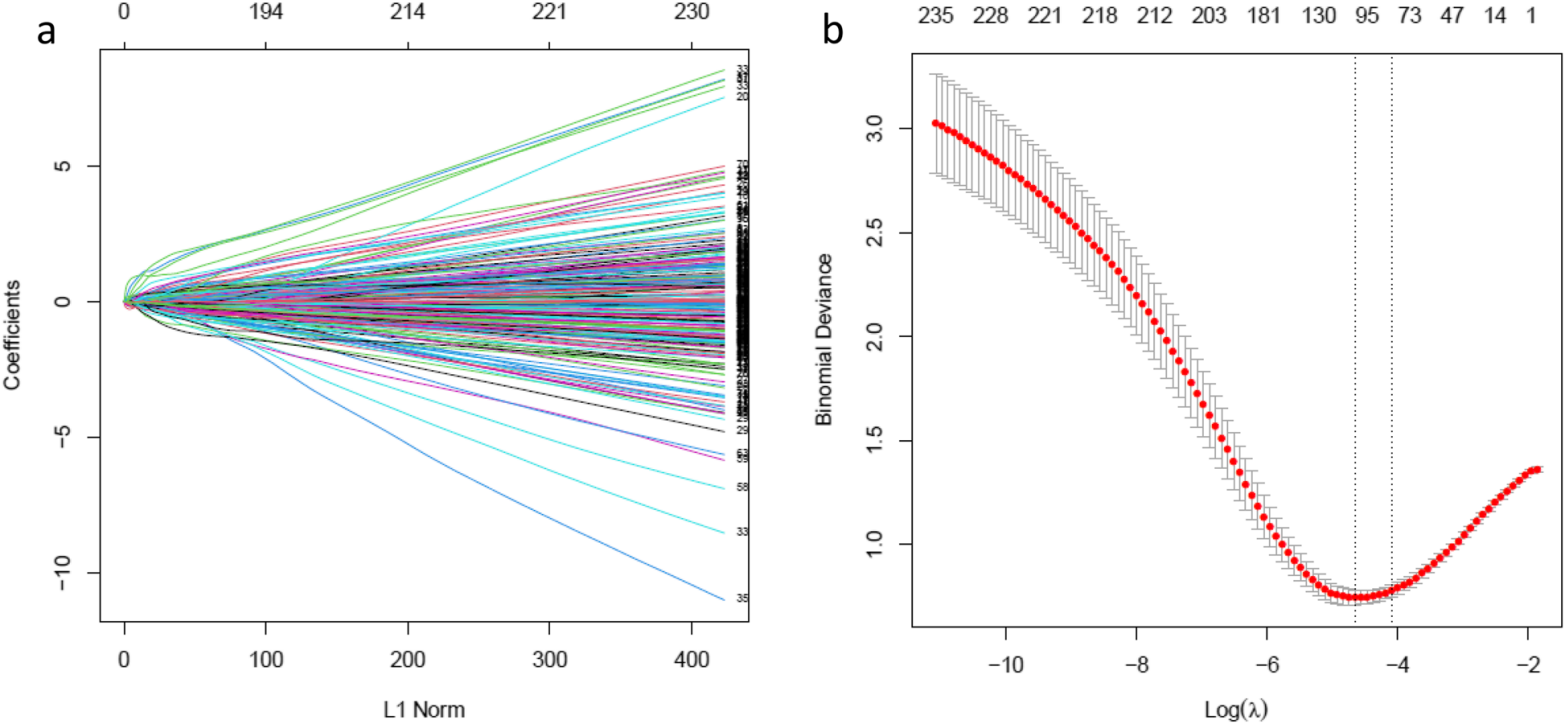
Genes were screened by Least absolute shrinkage and selection operator (LASSO) regression analysis (**a**). The LASSO model and cross validation method were used to screen genes. Cross validation plot indicated when the number of variables was 104, the partial likelihood deviation was the minimum (**b**). Dotted vertical lines were drawn at the optimal values by using the minimum criteria and the 1 standard error (SE) of the minimum criteria

Using LASSO on the gene expression matrix of 353 DEGs, a set of 104 genes was identified (Fig. 5). The gene list of 104 selected genes has been reported in Supplementary Sheet 3.

### Correlation-based feature selection

Employing CFS with a correlation threshold of 1, a set of 10 genes was identified. The discriminative genes selected through CFS are GI_10092618-S-4, GI_10835022-S-8, GI_37059795-S-1, GI_37059795-S-5, GI_46255021-A-6, GI_51477209-S-8, GI_53729348-S-2, GI_56119169-S-5, GI_71773149-A-8, GI_9945331-S-4 (NFKBIA, ITPR1, MGC26963, MGC26963, ERG, BEXL1, PLAU, CCL2, APRT, and GADD45B, respectively).

### Validation based on the high-correlated modules

**Fig. 6:**
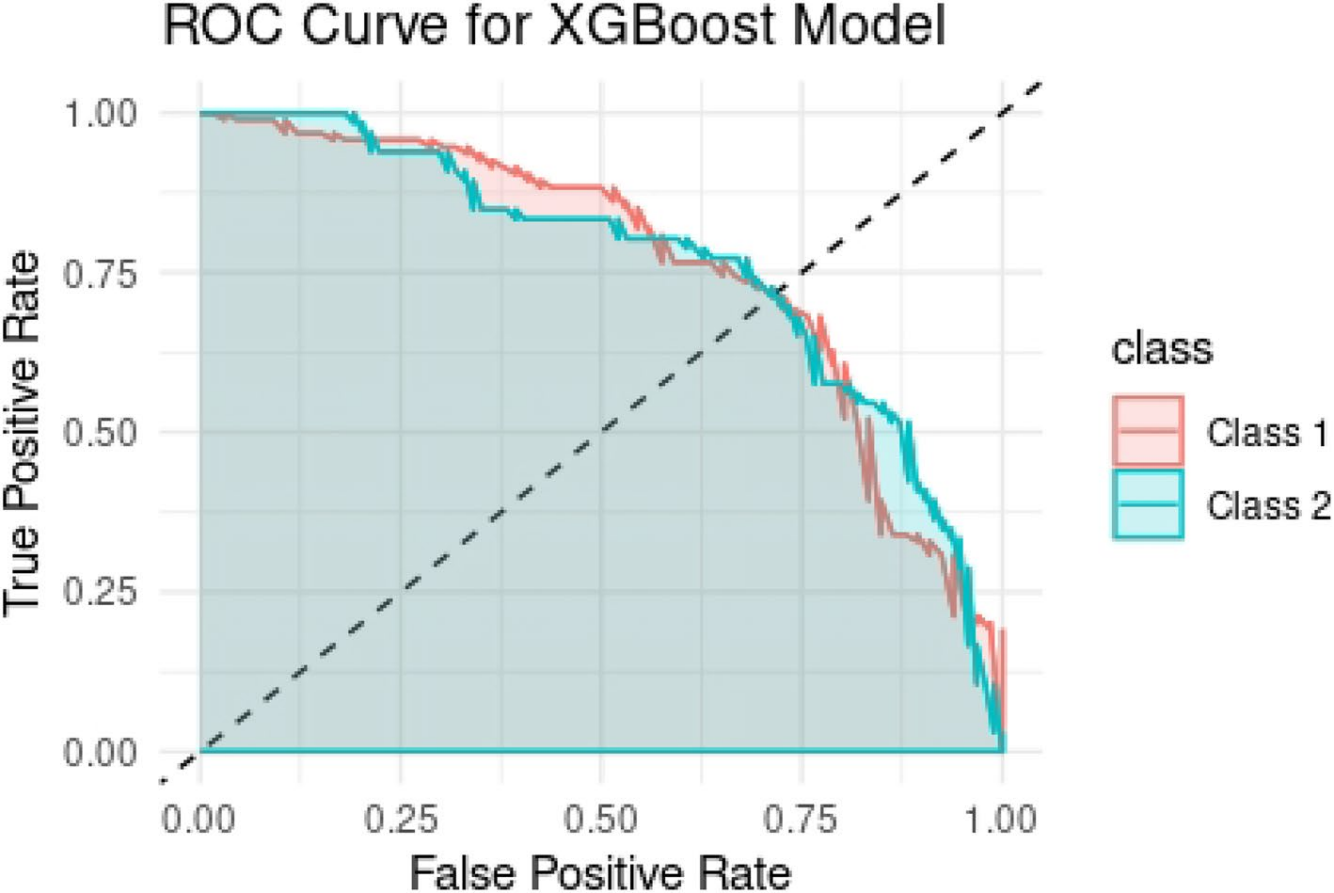
ROC curve analysis to test the validity of gene expression of high-correlated modules in discriminating European-American Men (EAM) and African-American Men (AAM) samples

**Fig. 7:**
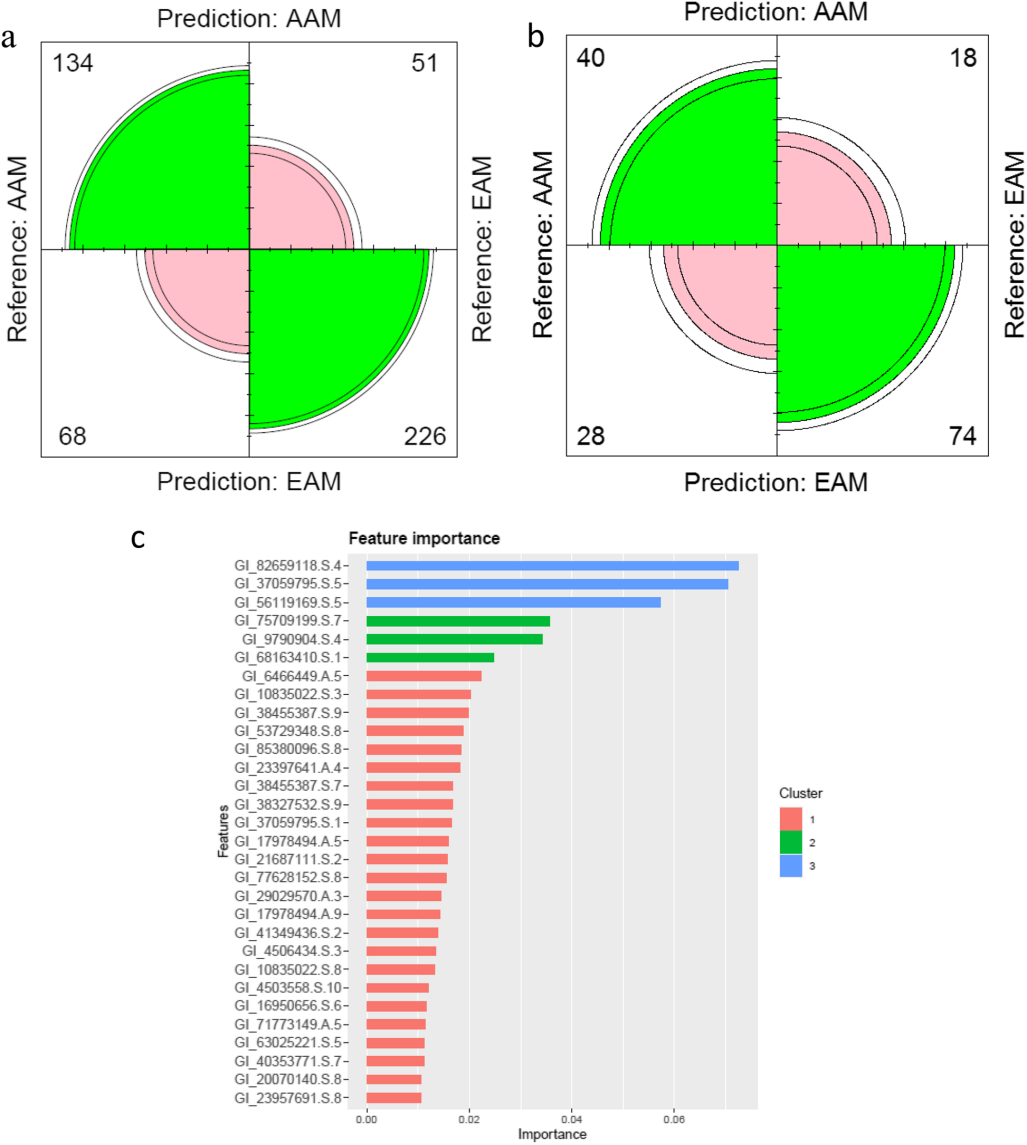
Confusion matrices for XGBoost, performed on the Out-of-fold (OOF) predicted class probabilities in the training data (**a**) on the test data (**b**). Importance ranking of top-30 genes affecting prostate cancer occurrence derived from applying the XGBoost model predictions based on the blue and yellow modules identified through weighted gene co-expression network analysis (WGCNA) (**c**)

In our experiment, we performed a comparison of the XGBoost classifier, setting the parameters of XGBoost as default (learning_rate = 0.3, gamma = 0, max_depth = 6, and λ = 1). The number of rounds and cv.nfold were set to 50 and 5, respectively. Table 1 gives the performance of XGBoost results for validation in train and test samples. The model archives 0.7516 and 0.7125% prediction accuracy in train and test sets, respectively, indicating satisfactory results. The ROC curve for validation based on the high-correlated modules in discriminating European-American Men (EAM) and African-American Men (AAM) samples can be observed in Fig. 6. Clearly, the XGBoost classifier performs much better in sensitivity and recall compared to the other parameters (Table 1). The confusion matrix for XGBoost, performed on the out-of-fold (OOF) predicted class probabilities in the training data and test data, is provided in Fig. 7a and b, respectively. Importance ranking of the genes in blue and yellow modules was performed, since these two modules exhibited a strong correlation with pathways involved in prostate cancer. The top 30 genes affecting prostate cancer based on the blue and yellow modules are presented in Fig. 7c.

**Table 1:** Comparison of performance metrics for the XGBoost classifier to distinguish European-American Men (EAM) and African-American Men (AAM)

Parameters	train	Test
Accuracy	0.7516	0.7125
Sensitivity	0.8159	0.8043
Specificity	0.6634	0.5882
Pos Pred Value	0.7687	0.7255
Neg Pred Value	0.7243	0.6897
Precision	0.7687	0.7255
Recall	0.8159	0.8043
F1	0.7916	0.7629
Prevalence	0.5783	0.5750
Detection Rate	0.4718	0.4625
Detection Prevalence	0.6138	0.6375
Balanced Accuracy	0.7396	0.6963

## Discussion

The dendrogram identifies six modules based on the similarity of expression patterns among their genes. The modules are denoted by various colors (e.g., MEyellow, MEblue, MEturquoise, etc.), and their association with the population groups is indicated by numerical values and significance levels (Fig. 4). Based on the analysis of the link between modules and traits, a significant positive or negative association with either group is observed. A positive number signifies that the genes within this module have more expression in AAM compared to EAM. A negative number signifies that the genes within the module have lower levels of expression in AAM compared to EAM.

This implies that the genes inside these modules exhibit distinct behavior among different population groups, indicating the presence of biological processes or mechanisms in cancer that are affected by these difference. These modules are crucial for comprehending the distinct manifestation or progression of cancer in various populations.

The blue and yellow modules exhibit a strong correlation with the prostate cancer pathway, suggesting that the genes within these modules might contribute to the development and progression of prostate cancer. By prioritizing the modules that are enhanced in prostate cancer pathways, we may potentially discover crucial genes and molecular interactions that are unique to the disease. These findings could serve as possible biomarkers for diagnosis or targets for treatment. Conducting functional tests to validate the functions of these genes and pathways in prostate cancer is crucial.

A total of 104 genes were discovered using LASSO. Using CFS with a correlation threshold of 1, a group of 10 genes was subsequently found. Notably, six genes were found to be common between these two approaches. The precision rate of the six shared genes achieved a level of 73%. The identification of these six genes, through the combined utilization of LASSO and CFS techniques, signifies a momentous advancement in comprehending the essential genetic elements that contribute to prostate cancer. The presence of these often-found genes suggests a significant connection to the studied condition, as they are supported by both research methods. Additional investigation and examination of these genes may provide a crucial understanding of their functional roles, pathways, and their significance in relation to the causes, prognosis, or therapy approaches for prostate cancer. Furthermore, exploring the regulatory networks or interactions involving these genes could reveal new insights into comprehending the process of the disease.

Adenine phosphoribosyltransferase (APRT) is a metabolic enzyme that participates in the production of polyamines, which are essential for the rapid growth of cancer cells. APRT (Adenine Phosphoribosyltransferase) has the potential to be a target for cancer treatment, as suppressing the APRT gene has harmful effects on leukemia cell lines [18].

CCL2 is involved in the onset and advancement of several types of malignancies. It can stimulate the growth and multiplication of tumor cells through various mechanisms and facilitate the migration of cancer cells. Additionally, it can attract cells that inhibit the immune system to the surrounding environment of the tumor, thereby promoting the progression of cancer [19]. CCL2 is the most potent chemoattractant in the tumor microenvironment, responsible for attracting macrophages and initiating inflammation. It exerts chemotactic effects on neighboring host cells inside the tumor microenvironment and collaboratively influences their differentiation with other cytokines. Nevertheless, the presence of CCL2 in tumor patients leads to a detrimental impact on their prognosis, as it leads to the buildup of cell subtypes that suppress the immune system [20]. In addition, CCL2 attracts immune cells, specifically monocytes and macrophages, which subsequently transform into immunosuppressive myeloid-derived suppressor cells (MDSCs) and M2 macrophages. This recruitment worsens the immunosuppressive tumor microenvironment and undermines the effectiveness of treatment. In their 2021 study, Liu and colleagues discovered that CCL2 is the primary mediator released by tumor-associated adipocytes into the surrounding extracellular environment. They also developed a protein trap that effectively binds to CCL2 with strong affinity and specificity, allowing for the manipulation of CCL2-mediated immune responses. This approach demonstrated improved treatment effectiveness and significant suppression of tumor development [21].

BEX2 and its homolog BEX1 have a strong correlation in their expression and are members of a cluster that is enriched with genes involved in the ER response and apoptosis. The gene BEX2 has been recognized as being expressed at higher levels in a specific group of breast tumors that have estrogen receptors (ER). Additionally, it has been linked to better results following treatment with tamoxifen [22]. Nevertheless, there is a lack of explicit data about the involvement of BEXL1 in cancer.

MGC26963, alternatively referred to as Sphingomyelin synthase 2 (SGMS2), is a genetic element that has been associated with multiple forms of cancer. Research has demonstrated a significant association between the expression of SGMS2 mRNA and the presence of tumor-associated macrophages (TAMs), as well as a negative impact on the prognosis of patients with pancreatic ductal adenocarcinoma (PDAC) [23]. High levels of M2-polarized macrophages in the original tumor of triple-negative breast cancer (TNBC) are linked to a dismal prognosis. Blocking SGMS2 or genetically eliminating its expression decreases the M2 polarization of tumor-associated macrophages and hinders the advancement of tumors in triple-negative breast cancer (TNBC) [24]. Ovarian cancer exhibits a unique upregulation of SMS2, which actively promotes the migration, development, and survival of cancer cells. Suppression of SMS2 by depletion or inhibition hinders the migration, development, and survival of ovarian cancer cells [25]. SGMS2 enhances the growth and spread of cancer cells in breast cancer by utilizing a mechanism connected with ceramide and activating the TGF-β/Smad signaling pathway [26]. Using a mouse model, the absence of SMS2 hinders the development of the tumor microenvironment and prevents the entry of cancer cells [27].

PLAU, the urokinase-type plasminogen activator, exerts a substantial influence on the advancement of cancer. It facilitates cell growth, movement, attachment, and various other activities using the proteolytic system, intracellular signal transmission, and chemokine activation [28]. Increased PLAU expression is linked to heightened aggressive characteristics, stromal score, and immune suppression in pancreatic ductal adenocarcinoma (PDAC) [29]. PLAU is additionally linked to the movement and infiltration of cells and is controlled by the transcription factor YY1 in cervical cancer [30]. In addition, PLAU, sometimes referred to as a urokinase-type plasminogen activator (uPA), stimulates the movement, infiltration, and multiplication of colorectal cancer cells through the Src/ERK pathway [31]. Hence, directing efforts towards PLAU could potentially yield diagnostic, prognostic, and therapeutic benefits in many cancer types [32].

Remarkably, the analysis of both the LASSO and CFS approaches has led to the detection of six probes, with four of them located within the yellow module. The significance of the yellow module in the context of prostate cancer research is emphasized by this association. Furthermore, it has been noted that the genes in the yellow module demonstrate elevated levels of expression in AAM in comparison to EAM. This indicates a possible gene expression pattern that is specific to certain populations, which could have significant ramifications for the susceptibility to diseases and prognosis.

We employed Weighted WGCNA to investigate the genetic characteristics of prostate cancer across various population groups. The modules revealed in the investigation of the link between modules and traits reveal a clear gene expression pattern that is associated with different population backgrounds. These findings indicate that population factor (AAM vs. EAM) have a certain degree of influence on the genetic basis of prostate cancer. The genes found by LASSO (Linkage Analysis of Sequence Outliers) and CFS (Correlation-based Feature Selection) provide promising targets for comprehending the molecular mechanisms underlying these differences.

The discovery of genetically related modules in prostate cancer that are associated with race is consistent with prior studies that have demonstrated variances in genes among different races. Research has indicated that African-American men have a greater occurrence and severity of prostate cancer, possibly due to the varying activity of specific genes. Our research emphasizes particular gene clusters and genes that may play a crucial role in these variances.

Further work is necessary for the noteworthy modules and genes. Their prominent position in the gene networks implies that they could be crucial catalysts for the biological processes linked to disparities in prostate cancer. The Gene Ontology study offered further context by establishing connections between these genes and distinct cellular processes and molecular activities, thus enhancing our overall comprehension of their potential influence.

These discoveries create opportunities for more focused genomic investigations and potentially individualized therapeutic approaches. Gaining insight into the genetic determinants responsible for disparities in prostate cancer among different population groups may result in the development of more efficient screening, diagnosis, and treatment procedures that are customized for distinct populations. Moreover, including these genetic markers in clinical trials has the potential to advance the creation of treatments that are very efficient in many populations. Although our study offers valuable insights, it does have limits. Dependence on publicly accessible microarray datasets can lead to biases and limit the generalizability of the findings to all population groups. Subsequent investigations should prioritize the verification of these discoveries via clinical trials and broaden the scope of the analysis to encompass a more extensive range of genetic information. Incorporating environmental and lifestyle factors could provide a comprehensive perspective on the underlying causes of differences in prostate cancer.

Ultimately, our study emphasizes the significance of taking population characteristics into account when conducting a genetic analysis of prostate cancer. The identified gene modules and genes offer a fundamental comprehension of the molecular variations that could potentially contribute to the reported discrepancies in prostate cancer occurrence and advancement among various population groups. This research not only contributes to current knowledge but also emphasizes the necessity for individualized approaches in cancer therapy and care.

Our study deepens significant differences in gene expression patterns of prostate cancer between African American men (AAM) and European American men (EAM). These findings may be essential to develop personalized diagnosis resulted in more effective therapeutic strategies. The identification of potential biomarkers such as APRT, CCL2, BEX2, MGC26963, and PLAU through specific gene modules and key genes could enhance our perception of prostate cancer's molecular mechanisms and targeted treatments. However, several limitations could introduce potential biases. The public microarray gene expression profile (GSE41967) from a single geographic location and timeframe may not be representative of other populations or current clinical settings. The lack of information on metastatic disease and tumor characteristics, as well as the focus exclusively on AAM and EAM groups, limits the broader applicability of the results. Lifestyle factors, environmental exposures, and socioeconomic status may significantly affect cancer risk and progression, however, they were not considered in this study. Furthermore, microarray technology, while robust, has limitations compared to newer sequencing technologies that may potentially affect the resolution and sensitivity of gene expression differences. As well, Analytical methods such as WGCNA, LASSO regression, and CFS, have inherent biases related to their algorithmic assumptions. Reliance on publicly available datasets may introduce biases associated with sample selection and original study designs. Future studies is suggested to incorporate more diverse populations, consider environmental factors and socioeconomic, use advanced genomic technologies, and validate findings with independent datasets to enhance the robustness and applicability of the results.

## Conclusions

This study employed Weighted Gene Co-expression Network Analysis (WGCNA) to examine the gene expression patterns in cancer among two different population groups. Our thorough examination yielded valuable knowledge about the genetic foundations of cancer in these varied groups.

We identified gene modules that exhibit a substantial association with population characteristics. It is crucial to take into account population variety when studying the genetic foundation of cancer. The modules indicate the existence of population-specific biological pathways, which may be essential for customized medical strategies.

The results of this study emphasize the need to include population diversity in genomic research, specifically in the context of cancer studies. Gaining insight into the distinct and common genetic elements among different population groups can assist in the creation of improved, customized therapies and preventative measures. Additional investigation should prioritize the examination of the biological pathways and potential therapeutic targets found within the gene modules specific to the given population group. This has the potential to result in significant advancements in comprehending the mechanisms by which cancer originates and advances in various populations.

Although our work offers valuable insights, its scope is constrained by the gene expression data and the specific population groups that were examined. Subsequent investigations should encompass a wider spectrum of population groupings and incorporate supplementary genetic data types to achieve a more all-encompassing comprehension. By incorporating a larger sample size and incorporating other cancer kinds, we can enhance the credibility of our findings and gain a more comprehensive insight into the influence of population characteristics on cancer genetics.

To summarize, our research highlights the intricate nature of cancer genetics and the crucial influence of population variation on the formation of gene expression profiles. The findings obtained from this research establish the foundation for implementing more individualized and efficient strategies in the areas of cancer diagnosis, treatment, and prevention, specifically designed to cater to the distinct genetic composition of various population groups.

### Supplementary Information

Supplementary Material 1.

## Data Availability

No datasets were generated or analysed during the current study.
